# Inhibition of microRNA function by antimiR oligonucleotides

**DOI:** 10.1186/1758-907X-3-1

**Published:** 2012-01-09

**Authors:** Jan Stenvang, Andreas Petri, Morten Lindow, Susanna Obad, Sakari Kauppinen

**Affiliations:** 1 Institute of Veterinary Disease Biology, Faculty of Life Sciences, University of Copenhagen, DK-1870 Frederiksberg, Denmark; 2 Santaris Pharma, Kogle Allé 6, DK-2970 Hørsholm, Denmark; 3 Copenhagen Institute of Technology, Aalborg University, Lautrupvang 15, DK-2750 Ballerup, Denmark

## Abstract

MicroRNAs (miRNAs) have emerged as important post-transcriptional regulators of gene expression in many developmental and cellular processes. Moreover, there is now ample evidence that perturbations in the levels of individual or entire families of miRNAs are strongly associated with the pathogenesis of a wide range of human diseases. Indeed, disease-associated miRNAs represent a new class of targets for the development of miRNA-based therapeutic modalities, which may yield patient benefits unobtainable by other therapeutic approaches. The recent explosion in miRNA research has accelerated the development of several computational and experimental approaches for probing miRNA functions in cell culture and *in vivo*. In this review, we focus on the use of antisense oligonucleotides (antimiRs) in miRNA inhibition for loss-of-function studies. We provide an overview of the currently employed antisense chemistries and their utility in designing antimiR oligonucleotides. Furthermore, we describe the most commonly used *in vivo *delivery strategies and discuss different approaches for assessment of miRNA inhibition and potential off-target effects. Finally, we summarize recent progress in antimiR mediated pharmacological inhibition of disease-associated miRNAs, which shows great promise in the development of novel miRNA-based therapeutics.

## Introduction

MicroRNAs (miRNAs) are an abundant class of small (approximately 22 nt) endogenous non-coding RNAs that direct post-transcriptional regulation of gene expression. Metazoan miRNAs regulate a wide range of biological processes, including developmental timing, apoptosis, differentiation, cell proliferation and metabolism [1-6]. Moreover, there is ample evidence that dysregulation of individual or entire families of miRNAs is associated with the pathogenesis of human diseases, such as cancer, CNS disorders, viral infections, cardiovascular and metabolic diseases [7-12].

**Figure 1:**
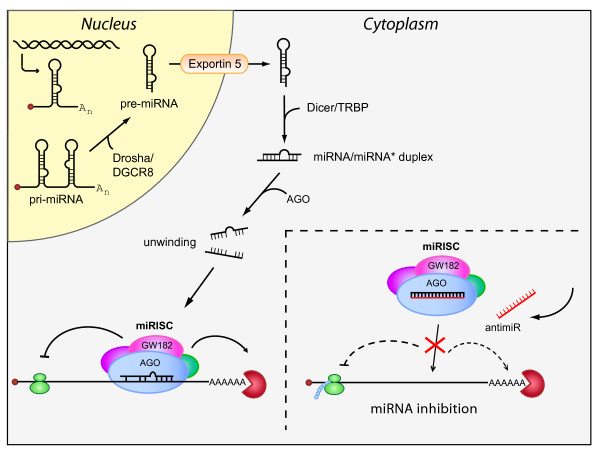
**miRNA biogenesis and inhibition of miRNA function by antimiR oligonucleotides**. miRNA genes are transcribed by RNA polymerase II into long primary miRNA transcripts, termed pri-miRNAs that are usually several kilobases long and possess a 5' CAP and a poly(A) tail. Pri-miRNAs are processed in the nucleus to ~70 nt pre-miRNAs by the nuclear Microprocessor complex, consisting of DGCR8 and the RNase III enzyme Drosha. Pre-miRNAs are exported to the cytoplasm by Exportin-5 and processed further by Dicer, to ~22 nt double-stranded miRNA duplexes that are loaded into an Argonaute protein in the miRISC and rapidly unwound. During this process the mature miRNA is retained in the miRISC, whereas the complementary strand, known as the miRNA star (miR*) is released. Metazoan miRNAs guide the miRISC to partially complementary sites in the 3' UTRs of target mRNAs to promote their translational repression or deadenylation and degradation. Chemically modified antimiR oligonucleotides sequester the mature miRNA in competition with cellular target mRNAs leading to functional inhibition of the miRNA and derepression of the direct targets.

The first miRNA genes, *lin-4 *and *let-7*, were discovered in *C. elegans *by Victor Ambros and Gary Ruvkun, and shown to base-pair imperfectly to 3' untranslated regions (UTRs) of heterochronic genes, thereby controlling timing of larval development in the worm [13-15]. To date 18,226 miRNAs have been annotated in animals, plants and viruses, including 1,527 miRNAs encoded in the human genome [16]. miRNAs are either expressed from independent transcriptional units or derive from introns of protein-coding genes or exons or introns of long ncRNAs. Approximately 50% of the mammalian miRNAs are located within introns of protein-coding genes [17,18]. The primary transcripts of miRNA genes, termed pri-miRNAs, are usually several kilobases long and possess a 5' CAP and a poly(A) tail [19,20]. Pri-miRNAs are processed in the nucleus to approximately 70 nt hairpin-structures, known as pre-miRNAs (Figure 1), by the nuclear Microprocessor complex, consisting of DGCR8 and the RNase III enzyme Drosha [21-23]. Pre-miRNAs are exported to the cytoplasm by Exportin-5 [24-27] and processed further by Dicer, to approximately 22 nt double-stranded miRNA duplexes (Figure 1) [28-32]. The miRNA duplexes are loaded into an Argonaute protein in the miRNA-induced silencing complex (miRISC) and rapidly unwound. During this process the mature miRNA is retained in the miRISC, whereas the complementary strand, known as the miRNA star (miR*), is released [33,34].

Most metazoan miRNAs guide the miRISC to partially complementary sites located in the 3' UTRs of target mRNAs, and, thereby, promote their translational repression or deadenylation and degradation [34,35]. A key specificity determinant for miRNA target recognition is based on Watson-Crick pairing of the so-called seed region (nucleotides 2 to 8) in the mature miRNA to the seed match site in the target 3' UTR, which nucleates the miRNA:target mRNA interaction [36]. The number of targets has been predicted using genome-wide computational searches for conserved seed match sites in mammalian 3' UTRs, which together with additional 3'-supplementary and 3'-compensatory binding sites imply that miRNAs may repress more than 60% of all mammalian protein-coding genes [36,37].

Identification and experimental validation of miRNA targets is a key prerequisite for uncovering the widespread biological roles of miRNAs and miRNA-mediated gene regulatory networks. This has accelerated the development of several computational, biochemical, genetic and functional genomics approaches for miRNA studies [36,38-44]. The most commonly used gain- and loss-of-function strategies to probe miRNA functions *in vitro *and *in vivo *are described in Table 1.

**Table 1:** Strategies to manipulate microRNA activity for gain- and loss-of-function studies

Loss-of-function				
**Technology**	**Characteristics**	***In vitro ***	***In vivo ***	**References**

Genetic knockout animals	Constitutive or conditional	Primary cells	Systemic or organ-specific	[43]
miRNA sponges	Transient to long-term inhibition	Transfection or viral delivery	Lentivirus or AAV-mediated delivery	[45-47]
antimiR oligonucleotides	Transient (*in vitro*) to long-lasting inhibition	Transfection or unassisted uptake	Unconjugated or 3'-cholesterol modified, i.v., s.c. or i.p. delivery	[48-50]
Target protectors	Transient	Transfection	Embryo injection (zebrafish)	[51-54]

**Gain-of-function**				

**Technology**	**Characteristics**	***In vitro ***	***In vivo ***	**References**

Transgenic animals	Constitutive or conditional	Primary cells	Systemic or organ-specific	[55,56]
Synthetic miRNA mimics	Transient	Transfection	Intratumoral injection, *ex vivo *transfection of cells, i.v. delivery	[57-62]
Vector-mediated miRNA over-expression	Transient to long-term over-expression	Transfection or viral delivery	Lentivirus or AAV-mediated delivery, intranasal delivery	[58-60,63,64]

Currently, three approaches are used in miRNA loss-of-function studies: genetic knockouts, miRNA sponges and antisense oligonucleotides. Knockout mice lacking key miRNA processing factors, such as Dicer, Drosha and Ago2, are embryonic lethal, which demonstrates the general importance of miRNAs in early embryonic development [43]. Generation of miRNA gene knockouts has been extensively used to unravel the functions of miRNAs in *C. elegans *and *Drosophila*, and has also been reported for many individual miRNAs in the mouse [8,10,65-71]. Recently, a genome-wide miRNA knockout resource covering 476 mouse miRNA genes was described, and has now been made available from ES cell repositories for distribution to the scientific community [72]. The use of miRNA sponges, which are highly expressed transgenes harboring multiple miRNA target sites to sequester miRNAs, represents another strategy to modulate miRNA activity for loss-of-function studies [73]. This approach enables both transient and long-term inhibition of entire miRNA seed families in cultured cells and has also been applied to manipulate miRNA activity in *Drosophila *and mice (reviewed by Ebert and Sharp [45]). Interestingly, systemic administration of a lentiviral sponge for miR-326 was shown to reduce the number of IL-17 secreting Th-17 cells and ameliorate experimental autoimmune encephalomyelitis (EAE) in mice [47]. These findings highlight the potential of virally delivered miRNA sponges in the development of miRNA-based gene therapies.

A widely employed approach in miRNA loss-of-function studies is to use chemically modified antisense oligonucleotides, termed antimiRs, which sequester the mature miRNA in competition with cellular target mRNAs leading to functional inhibition of the miRNA and derepression of the direct targets (Figure 1). Here, we describe the current designs of chemically modified antimiR oligonucleotides and provide an overview of antimiR *in vivo *delivery strategies. In addition, we discuss the assessment of miRNA inhibition and off-target effects, as well as the utility of antimiR compounds in pharmacological inhibition of disease-implicated miRNAs for therapeutics.

## Design of chemically modified antimiR oligonucleotides

**Figure 2:**
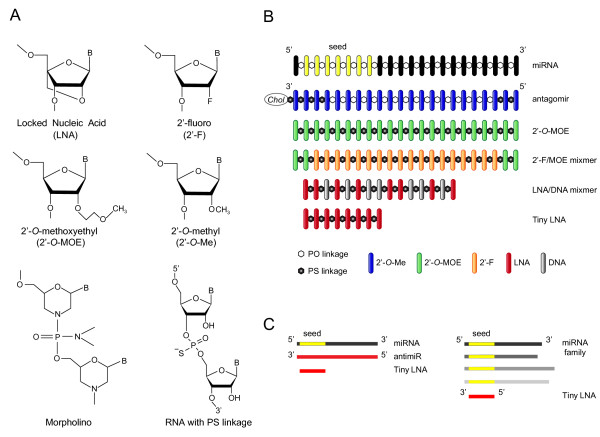
**Design of chemically modified antimiR oligonucleotides**. **(A) **Structures of the most commonly used chemical modifications in antimiR oligonucleotides. Locked nucleic acid (LNA) is a bicyclic RNA analogue in which the ribose is locked in a C3'-endo conformation by introduction of a 2'-O,4'-C methylene bridge. The 2'-fluoro (2'-F), 2'-*O*-methoxyethyl (2'-MOE) and 2'-*O*-methyl (2'-*O*-Me) nucleotides are modified at the 2' position of the ribose moiety, whereas a six-membered morpholine ring replaces the sugar moiety in morpholino oligomers. In the phosphorothioate (PS) linkage, sulfur replaces one of the non-bridging oxygen atoms in the phosphate group. **(B) **Design of chemically modified antimiR oligonucleotides described in this review. **(C) **Schematic overview of the miRNA inhibition approach using a fully complementary antimiR and a seed-targeting tiny LNA.

MiRNA inhibition by antimiRs requires optimization of the oligonucleotides for increased binding affinity, improved nuclease resistance and *in vivo *delivery. This can be achieved using a variety of chemical modifications, including modifications of the sugar, the nucleobase or the internucleotide linkages (Figure 2A, B). Sequence-specific inhibition of miRNA function was first demonstrated in cultured HeLa cells using 2'-*O*-methyl (2'-*O*-Me) modified RNA oligonucleotides complementary to mature miRNAs [74,75]. The 2'-*O*-Me modification as well as the 2'-*O*-methoxyethyl (2'-MOE) and 2'-fluoro (2'-F) chemistries are modified at the 2' position of the sugar moiety, whereas locked nucleic acid (LNA) comprises a class of bicyclic RNA analogues in which the furanose ring in the sugar-phosphate backbone is chemically locked in a RNA mimicking N-type (C3'-endo) conformation by the introduction of a 2'-O,4'-C methylene bridge (Figure 2A) [50,76-80]. All the aforementioned modifications confer nuclease resistance and increase the binding affinity of antimiR oligonucleotides to their cognate miRNAs. Among these, LNA possesses the highest affinity towards complementary RNA with an increase in duplex melting temperature (T_m_) of +2 to 8°C per introduced LNA monomer against complementary RNA compared to unmodified duplexes [80-83]. Another important observation is that LNA monomers are also able to twist the sugar conformation of flanking DNA nucleotides from an S-type (C2'-endo) towards an N-type sugar pucker in LNA-modified DNA oligonucleotides [80,84]. Indeed, structural studies of different LNA-RNA and LNA-DNA heteroduplexes based on NMR spectroscopy and X-ray crystallography have shown that LNA-modified DNA oligonucleotides are RNA mimics, which fit seamlessly into an A-type Watson-Crick duplex geometry [84,85] similar to that of dsRNA duplexes.

Nuclease resistance is also improved by backbone modification of the parent phosphodiester linkages into phosphorothioate (PS) linkages in which a sulfur atom replaces one of the non-bridging oxygen atoms in the phosphate group (Figure 2A) or by using morpholino oligomers, in which a six-membered morpholine ring replaces the sugar moiety. Morpholinos are uncharged, inherently resistant to degradation by nucleases and exhibit only a slight increase in binding affinity to miRNAs [86]. Morpholino oligomers have been shown to be sequence-specific, non-toxic and potent inhibitors of both pri-miRNA and mature miRNA activity in zebrafish and *Xenopus laevis *[87-89].

Several studies have evaluated the potency of different chemically modified antimiR oligonucleotides in miRNA inhibition [76,77,90-93]. Two studies used luciferase reporter assays to compare different antimiR designs in targeting of miR-21 in HeLa cells. Davis *et al. *[76] showed a loose correlation between binding affinity and *in vitro *antimiR potency and found that among the fully PS modified antimiRs investigated, those with the highest T_m_, a uniform 2'F and a LNA/2'-MOE mixmer antimiR, were the most potent miR-21 inhibitors. Similarly, Lennox and Behlke [92] reported that incorporation of high affinity modifications to antimiR oligonucleotides improved their potency. In their study, LNA/2'-*O*-Me mixmers with PS ends or with a complete PS backbone showed highest potency, being approximately 10 times more potent than a uniform 2'-*O*-Me modified antimiR. Consistent with these observations, we found that inhibition of miR-122 function in cultured Huh-7 cells by different LNA/DNA mixmers was affinity dependent and identified a LNA-modified antimiR with a high T_m _of 80°C, which mediated efficient de-repression of a miR-122 luciferase reporter upon co-transfection of the antimiR-122 into Huh-7 cells at 5 nM concentration [90]. Moreover, this antimiR-122 was also the most potent inhibitor of HCV RNA accumulation in Huh-7 cells harboring the HCV-N replicon, compared with a 2'-*O*-Me oligonucleotide and two LNA-antimiRs of lower affinity [90]. Effective targeting of miR-122 by LNA/2'-*O*-Me and 2'-F/MOE modified antimiRs (Figure 2B), respectively, has also been reported [77,94]. In most studies to date, fully complementary antimiRs have been used to target the mature miRNA. Notably, truncation of a uniform 2'-MOE-modified antimiR-21 and a cholesterol-conjugated antagomir-122, respectively, by three or more nucleotides was shown to result in substantial or complete loss of efficacy in cultured cells and *in vivo *[76,95]. By comparison, we and others have reported on efficient antagonism of several miRNAs, using high-affinity 15 to 16 nucleotide LNA-modified DNA/PS oligonucleotides targeting the 5' region of the mature miRNA (Figure 2B) [11,90,96-102]. Furthermore, we have recently described a method that enables inhibition of miRNA function using short seed-targeting LNA oligonucleotides, designated as tiny LNAs [103]. This approach exploits the high binding affinity of fully LNA-modified 8-mer PS oligonucleotides complementary to the miRNA seed region (Figure 2B, C), which enables specific and concentration-dependent inhibition of entire miRNA seed families in cultured cells with concomitant de-repression of direct targets [103]. Our data highlight the importance of targeting the miRNA seed for inhibition, as 8-mer LNAs targeting other regions in mature miRNA sequences had no or limited effect on miRNA activity. The importance of the high binding affinity of fully substituted 8-mer LNAs was further demonstrated by the fact that an 8-mer 2'-*O*-Me-modified antimiR-21 oligonucleotide with a low T_m _of 37°C showed no inhibition of miR-21 activity in HeLa cells [103].

## *In vivo *delivery of antimiR oligonucleotides

Inhibition of miRNA function *in vivo *was first described in *C. elegans *by Hutvágner *et al. *[74]. In this study, a 2'-*O-*Me oligonucleotide complementary to let-7 was microinjected in *C. elegans *larvae and shown to phenocopy the let-7 loss-of-function mutation [74]. The utility of 3' cholesterol-conjugated, 2'-*O*-Me oligonucleotides with terminal PS modifications, termed antagomirs (Figure 2B), in pharmacological inhibition of miRNAs in mice, was described in 2005 by Krutzfeldt *et al. *[104]. Treatment of mice with three tail vein injections of 80 mg/kg antagomir-16 resulted in silencing of miR-16 in the liver, kidney, lung, heart, skeletal muscle, colon, fat, skin, ovaries, adrenal glands and bone marrow, whereas no efficacy was observed in the brain [104]. Furthermore, systemic delivery of antagomir-122 by three intravenous (i.v.) injections of 80 mg/kg led to efficient inhibition of the liver-expressed miR-122 with concomitant de-repression of liver mRNAs with miR-122 seed match sites and a 40% decrease in serum cholesterol levels in the treated mice [104]. Subsequent studies showed that systemically delivered antagomir-122 accumulates in a cytoplasmic compartment of hepatocytes distinct from P-bodies and inferred an antagomir-mediated degradation mechanism independent of the RNAi pathway [95]. Moreover, efficient inhibition of miR-16 in the brain was achieved by direct delivery of antagomir-16 in the mouse cortex [95].

PS backbone modifications greatly improve the pharmacokinetic properties of antisense oligonucleotides, thereby facilitating their delivery *in vivo *[105]. Indeed, several studies have reported efficient and long-lasting silencing of miRNAs *in vivo *using unconjugated 2'-F/MOE-, 2'-MOE- and LNA-modified antimiRs harboring a complete PS backbone (Figure 2B) [11,77,90,96,97,102,106-108]. We have described potent and specific miR-122 silencing *in vivo *using a high-affinity 15 nucleotide LNA/DNA mixmer PS oligonucleotide complementary to the 5' end of miR-122 [90]. Administration of unconjugated, saline-formulated LNA-antimiR-122 to mice either intraperitoneally (i.p.) or i.v. resulted in efficient uptake of the compound in the liver, which coincided with a dose-dependent sequestration of mature miR-122 in a highly stable heteroduplex with LNA-antimiR, inferring a different mode of action compared to the degradation mechanism described for antagomirs [95,104] and 2'-MOE-modified oligonucleotides [106]. Using single i.p. injections of the LNA-antimiR at doses ranging from 1 to 200 mg/kg we observed a dose-dependent lowering of serum cholesterol in mice with a median effective dose of 10 mg/kg, whereas treatment of high fat diet-fed mice with 5 mg/kg LNA-antimiR twice weekly for six weeks led to sustained lowering of serum cholesterol by 30% and de-repression of predicted target mRNAs with canonical miR-122 seed match sites [90]. Moreover, systemic administration of PBS-formulated LNA-antimiR to African green monkeys at doses ranging from 1 to 10 mg/kg with three i.v. infusions over five days resulted in accumulation of the LNA-antimiR compound in the liver and concomitant, dose-dependent sequestration of mature miR-122 in a shifted LNA-antimiR:miR-122 heteroduplex in Northern blots. This led to a dose-dependent and long-lasting decrease of serum cholesterol in the treated primates, which gradually returned to baseline levels over a three-month period after treatment. Importantly, the LNA-antimiR compound was well tolerated in both mice and primates as no acute or subchronic toxicities in the treated animals were detected [90].

In a recent study, we investigated whether the high binding affinity of seed-targeting tiny 8-mer LNA-antimiRs could enable delivery and silencing of miRNAs *in vivo *without additional conjugation or formulation chemistries when combined with a complete PS backbone [103]. A systemically delivered 8-mer antimiR-122 (three i.v. doses of 5 or 20 mg/kg) was shown to sequester miR-122 in the mouse liver, leading to concomitant de-repression of predicted miR-122 target mRNAs with canonical 3' UTR seed match sites and dose-dependent lowering of serum cholesterol, which is consistent with previous reports in rodents and non-human primates [90,103,104,106]. Furthermore, a systemically delivered^ 35^S-labeled tiny antimiR-21 showed uptake in many tissues in mice with high levels of the compound accumulating in the kidney cortex, liver, lymph nodes, bone marrow and spleen with terminal half-lives ranging from 4 to 25 days [103]. The terminal elimination half-life in heart blood was 8 to 10 hours and urine and bile were indicated as primary routes of elimination. The tiny antimiR-21 also sequestered the target miRNA in the liver, kidney and lung coinciding with up-regulation of the miR-21 target BTG2 in the same tissues [103]. These findings imply that tiny LNAs could become a useful tool for functional studies of animal miRNAs *in vivo*, since unlike other chemically modified antimiRs, 8-mer LNAs enable inhibition of co-expressed miRNA family members that may have redundant biological functions.

## Assessment of miRNA inhibition

**Figure 3:**
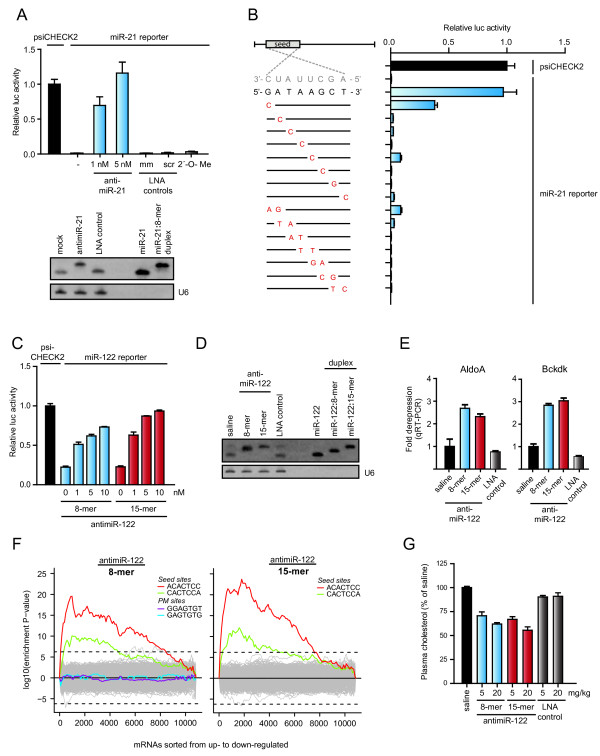
**Assessment of miRNA inhibition in cultured cells and *in vivo***. **(A) **Upper panel. Relative luciferase activity of the miR-21 reporter containing a perfect match miR-21 target site co-transfected into HeLa cells with 1 or 5 nM tiny LNA-antimiR-21, or 5 nM 8-mer 2'-*O*-Me antimiR-21, LNA mismatch (mm) or LNA scramble (scr) control oligonucleotides, respectively. Error bars represent s.e.m. Lower panel. Northern blot analysis of miR-21 in HeLa cells transfected with 5 nM antimiR-21 or LNA scramble control. U6 is shown as control. **(B) **Relative luciferase activity of the miR-21 reporter co-transfected into HeLa cells, with 5 nM tiny seed-targeting LNAs harbouring single or two adjacent mismatches at all possible nucleotide positions in the antimiR-21 sequence (highlighted in red). **(C) **Relative luciferase activity of a miR-122 reporter containing a perfect match miR-122 target site co-transfected into HeLa cells with pre-miR-122 and tiny 8-mer antimiR-122 or 15-mer antimiR-122. Error bars represent s.e.m. **(D) **Northern blot analysis of liver RNAs from mice after treatment with three intravenous doses of 20 mg/kg 8-mer antimiR-122, 15-mer antimiR-122 or LNA scramble control or with saline. The Northern blot was probed for miR-122 and U6. **(E) **Quantification of the AldoA and Bckdk target mRNAs (same samples as in D, normalized to GAPDH; error bars, s.e.m.; n = 5). **(F) **Sylamer analyses performed on microarray data from mouse liver RNAs after treatment with three intravenous doses of 20 mg/kg 8-mer antimiR-122 or 15-mer antimiR-122. Shown are Sylamer enrichment landscape plots for 7 nt sequence words. The highlighted words in the plots correspond to canonical miR-122 seed match sites and to perfect match binding sites for the 8-mer antimiR-122. **(G) **Total plasma cholesterol levels in mice treated with three intravenous injections of 8-mer antimiR-122, 15-mer antimiR-122 or LNA scramble control or with saline (error bars, s.e.m.; n = 5). Adapted from Obad *et al. *[103].

The effect of miRNA inhibition by antimiR oligonucleotides can be assessed by a variety of approaches. Most methods that directly measure changes in miRNA levels are hybridization based assays and associated with several possible caveats when antimiR mediated miRNA inhibition is assessed. First, the antimiR chemistry appears to dictate the fate of the targeted miRNA. High-affinity oligonucleotides, such as LNA/DNA, LNA/2'-*O*-Me and 2'-F/MOE modified antimiRs, respectively, sequester the targeted miRNA in a heteroduplex [77,90,96,102,103,107,109], whereas lower affinity oligonucleotides, such as 2'-*O*-Me and 2'-MOE modified antimiRs and cholesterol-conjugated 2'-*O*-Me antagomirs, promote miRNA degradation [77,104,106,109]. A recent study in *Drosophila *reported that extensive complementarity between Ago1-loaded miRNA and its target RNA can trigger tailing and 3'-to-5' exonucleolytic trimming of the miRNA causing a decrease in mature miRNA abundance [110]. Notably, miRNA tailing and trimming were also observed in HeLa cells transfected with antagomirs fully complementary to miR-16 and miR-21, respectively [110], which is consistent with antagomir-mediated degradation of miRNAs descibed previously in mice [104]. We and others have reported on detection of stable antimiR:miR heteroduplexes as slower-migrating bands on small RNA Northern blots (Figure 3) [90,96,102,103,107,109,111]. However, this can be technically challenging due to difficulties in the recovery and detection of the heteroduplexes [109]. Second, the presence of excess antimiR in the RNA sample irrespective of the mechanism of action may interfere with the detection step of the assay, for example, primer annealing or extension in miRNA-specific real-time qPCR. The observed miRNA reduction in the readouts from such experiments could, therefore, be misleading due to the antimiR masking effects in the assays. Finally, antimiRs may be released from subcellular compartments during tissue homogenization and RNA extraction, thereby facilitating hybridization between the antimiR and the target miRNA during sample preparation. To help circumvent these pitfalls, especially for small RNA Northern blot analysis, it has been suggested to use stringent denaturing conditions during electrophoresis [104], to increase the hybridization temperature [112], to use LNA detection probes [90,96,103,107,112] or to include a competitor probe with an identical sequence as the miRNA prior to electrophoresis to release the miRNA from the miR:antimiR duplex [77,109]. Taken together, due to the possible assay interference and technical difficulties in antimiR:miR heteroduplex recovery, evaluation of antimiR mediated inhibition of miRNA function by direct methods should be interpreted with some caution. Thus, we recommend that direct measurements of the targeted miRNA should always be accompanied with assessment of the functional effects after miRNA antagonism, as outlined in Figure 3.

Assays that measure a functional readout of miRNA modulation by antimiRs are often employed to provide substantial evidence for miRNA inhibition. These approaches include miRNA reporter assays, assessment of de-repression of direct targets by real-time qPCR, Western blot analysis and genome-wide transcriptional or proteomic analyses. A simple and very sensitive approach involves construction of a miRNA reporter that carries a single or multiple perfect match or bulged miRNA binding sites in the 3' UTR of a reporter gene, such as luciferase or GFP. This method has been extensively used in cultured cells to validate miRNA inhibition (Figure 3A) and also to compare the potency of different chemically modified antimiR designs [76,90,92,103,112]. Recently, a miR-21 luciferase reporter was used in a mouse mammary tumor model to monitor functional inhibition of miR-21 by a seed-targeting antimiR-21 *in vivo *[103]. The antimiR specificity is typically assessed using control oligonucleotides, either by introducing one or more mismatches in the antimiR sequence or by using a scrambled sequence oligonucleotide. The potencies of such control oligonucleotides are expected to be markedly reduced, as shown in Figure 3B, in which the specificity of an 8-mer LNA-antimiR-21 was assessed by introducing one or two adjacent mismatches at all possible nucleotide positions in an 8-mer antimiR-21 sequence. Ideally, both control reporters with mutated miRNA target sites and mismatched or scrambled oligonucleotides should be included as specificity controls when assessing inhibition of miRNA function by miRNA reporter constructs.

The mechanism of miRNA-mediated mRNA repression involves both mRNA deadenylation and degradation and translational repression (Figure 1) [34,35]. Recent reports on simultaneous genome-wide measurements of changes in mRNA and protein levels after perturbing miRNA levels showed good correlation between mRNA and protein levels [39,40]. Thus, an alternative or supplemental approach to employing miRNA reporter assays is to use the levels of direct target mRNAs and their encoded proteins as functional readouts of miRNA silencing. Western blots are readily used to demonstrate the effect of antimiR mediated inhibition by assessing target de-repression at the protein level [103,113-115]. However, the degree of miRNA target de-repression is often modest and several high throughput analyses following miRNA perturbation report changes in mRNA levels of only 33 to 35% [116,117]. Moreover, proteomic studies that detect the effect of miRNA modulation by measuring directly the protein levels have reported that the average changes are less than two-fold [39,40]. Hence, more sensitive methods, such as qPCR or ELISA, might be better suited to estimate antimiR effects on single direct targets, as shown in Figure 3E for two direct miR-122 target mRNAs in the mouse liver.

Given that a single miRNA has the potential to regulate hundreds of mRNA targets, high throughput methods that enable genome-wide transcriptional and proteomic profiling offer the opportunity to get a broad view of the effects of miRNA antagonism. Moreover, effects of perturbing the miRNA activity can be detected more robustly by assessing the combined effect on all the predicted direct targets. Thus, in the case where single target analysis is unable to find a significant effect, the simultaneous analysis of a large group of target mRNAs increases statistical power and can yield highly significant findings. Expression microarrays have been widely used in transcriptional profiling experiments and have also been employed by several studies to assess genome-wide transcriptional changes after modulation of miRNA activity in cultured cells and *in vivo *[37,39,40,90,103,107,118]. However, the recent development in massively parallel sequencing technologies has prompted many researchers to use RNA sequencing (RNA-Seq) for genome-wide expression analyses. RNA-Seq enables not only assessment of transcript levels with unprecedented accuracy and a broad dynamic range, but provides a detailed view of the entire transcriptome at a level that can provide important information on, for example, alternative splicing, RNA editing and other post-transcriptional modifications without the requirement of prior knowledge needed for microarray probe design [119]. RNA-Seq was recently used to compare genome-wide transcriptional and proteomic changes mediated by ectopic and endogenous miRNAs in human and mouse cells [116,117], and to assess antagomir-directed tailing and trimming of miRNAs in cultured HeLa cells [110].

A widely used method of testing the significance after modulation of miRNA activity on multiple targets is the Kolmogorov-Smirnov test, which asks whether the distribution of antimiR-mediated transcriptional changes observed for a set of target mRNAs is significantly different from a set of non-target mRNAs [37,39,40,90,103,107,118]. While this type of analysis is cut-off free, it requires *a priori *knowledge of miRNA targets and is, thus, biased by the choice of target prediction algorithm and by preselecting the miRNA targets for analysis. An alternative, more unbiased approach to analyzing the effect of miRNA perturbation on target mRNAs is to use the Sylamer algorithm [120] that uses expression changes measured after, for example, miRNA silencing to rank genes and subsequently test the occurrence of all possible sequence motifs of a given length relative to the sorted gene list. The landscape plots resulting from this type of analysis (Figure 3F) show the significance profiles of all sequence motifs of a given length across the sorted gene list, as shown in Figure 3F for two different LNA-modified antimiR oligonucleotides targeting miR-122.

## Assessment of off-target effects

The use of antimiR oligonucleotides as a tool in functional miRNA studies or as a therapeutic modality carries the inherent risk of affecting RNA species other than the intended miRNA target. Thus, understanding the effects of unwanted interactions between the antimiR oligonucleotide and endogenous nucleic acids is of key importance and when appropriate, application of this knowledge during the design of antimiR molecules can help minimize off-target effects. The fact that longer oligonucleotides have fewer perfectly matched complementary sites in the transcriptome than shorter is sometimes used to state that longer oligonucleotides are more specific. However, this only holds true if the hybridization stringency can be controlled. When antimiRs are used *in vivo*, their interactions with RNA molecules are bound to take place at a physiologically relevant temperature and at reduced stringency. Thus, interactions are likely to occur through imperfect base pairing similar to, for example, non-specific priming observed in PCR at low annealing temperatures. The incorporation of chemical modifications, such as LNAs, into antimiRs, has been shown to improve mismatch discrimination [121], but this increased complexity versus simple Watson-Crick base pairing rules makes it difficult to accurately predict interaction sites for fully complementary antimiRs.

Due to their small size, 8-mer seed-targeting antimiRs have many predicted perfectly complementary sites in the transcriptome. Hence, by using Watson-Crick base pairing rules we are able to identify a substantial number of mRNAs that can be considered as candidates for off-targets. However, this does not necessarily imply that such sites are indeed occupied in the cell, nor is it given that such interactions if they occur have functional consequences by affecting the levels of the bound mRNAs or their encoded proteins. To address these questions, we have recently reported an empirical approach in which transcriptional and proteomic profiling was applied to measure the effects of tiny 8-mer LNAs in cell culture and *in vivo *[103]. We first used the Sylamer algorithm [120] to ask which sequence motifs were associated with differentially expressed genes following tiny antimiR treatment. While the direct effect of tiny LNA mediated miRNA silencing was readily detected in all our experiments (Figure 3F and [103]) by significant overrepresentation of miRNA seed match sites in the up-regulated mRNAs, no enrichment of sequence motifs in mRNAs with tiny LNA complementary sites was detected. This implies that predicted off-targets are randomly distributed across the sorted gene lists, and that as a group, the predicted off-target mRNAs are not affected by the antimiR. Next, we used proteomic data to measure the effect of tiny LNAs on predicted off-targets to test whether potential binding of tiny LNAs to mRNAs could affect their translation. Our findings showed that the distribution of expression changes following tiny LNA mediated miRNA silencing differed significantly when miRNA targets were compared to non-targets, reflecting de-repression of direct targets. Contrary to the effect observed on the miRNA targets, the levels of proteins derived from mRNAs with tiny LNA complementary sites were not affected, implying that tiny LNAs do not have a general effect on predicted off-target interaction partners [103].

Another potential antimiR mediated off-target effect was recently reported by Khan *et al. *[122], who showed that transfection of miRNA mimetics or siRNAs into cells leads to effects on endogenous miRNA targets. These findings are consistent with a model in which the exogenous si/miRNA competes with the endogenous miRNA for miRISC and the consequent loss of available miRISC leads to abrogation of endogenous miRNA mediated regulation. Similar saturation-based effects could be detected when analyzing data sets from antimiR cell culture experiments, which implied that treatment with antimiRs leads to global effects on targets of other endogenous miRNAs. This is consistent with the notion that an antimiR oligonucleotide sequesters its cognate miRNA in the miRISC complex, thereby rendering it unavailable to other endogenous miRNAs. However, further experiments are needed to pinpoint the exact molecular mechanisms leading to the observed effects and to fully understand the ramifications these findings may have on endogenous miRNA function.

## Therapeutic targeting of disease-associated miRNAs

Manipulation of miRNA activity *in vivo *is of high interest due to the aberrant expression and implication of miRNAs in the pathogenesis of human diseases. The use of antimiR oligonucleotides to target disease-associated miRNAs is the most widely used approach to probe their functions *in vivo *and shows great promise in the development of novel miRNA-based therapeutics. Indeed, an increasing number of studies have reported successful therapeutic miRNA silencing in a variety of animal disease models using antimiR oligonucleotides (Table 2). This section highlights selected studies, in which either 3' cholesterol-conjugated antagomirs or unconjugated, chemically modified antimiRs harboring a complete PS backbone have been used to pharmacologically inhibit disease-associated miRNAs *in vivo*.

**Table 2:** Therapeutic targeting of selected disease-associated miRNAs *in vivo *using antimiR oligonucleotides

miRNA	Model	antimiR chemistry	Administration route	Phenotype	Reference
miR-122	Chow-fed mice	Cholesterol-conjugated 2'-*O*-Me antagomir	i.v.	Lowering of serum cholesterol	[104]
miR-122	DIO mice	2'-MOE	i.p.	Lowering of serum cholesterol	[106]
miR-122	Chow-fed mice	LNA	i.v.	Lowering of serum cholesterol	[96]
miR-122	DIO mice, African green monkeys	LNA	i.p., i.v.	Lowering of serum cholesterol	[90]
miR-122	HCV-infected chimpanzees	LNA	i.v.	Suppression of viremia	[107]
miR-10b	Mouse mammary tumor model	Cholesterol-conjugated 2'-*O*-Me antagomir	i.v.	Suppression of lung metastases	[123]
miR-132	Orthotopic mouse model of human breast carcinoma	2'-*O*-Me	Vessel-targeted nanoparticle delivery i.v.	Reduction of angiogenesis and tumor burden	[124]
miR-199b	Mouse model of heart failure	Cholesterol-conjugated 2'-*O*-Me antagomir	i.p.	Inhibition and reversal of cardiac hypertrophy and fibrosis	[125]
miR-328	Mouse model of atrial fibrillation	Cholesterol-conjugated 2'-*O*-Me antagomir	i.v.	Normalization of atrial fibrillation	[126]
miR-21	Mouse model of lupus	LNA	i.v., i.p.	Amelioration of autoimmune splenomegaly	[115]
miR-17-5p	Mouse xenograft model of neuroblastoma	Cholesterol-conjugated 2'-*O*-Me antagomir	Intratumoral injection	Inhibition of tumor growth	[127]
miR-103/107	DIO and ob/ob mice	Cholesterol-conjugated 2'-*O*-Me antagomir	i.v.	Improved glucose homeostasis and insulin sensitivity	[128]
miR-138	NOD/SCID mice with hydroxyapatixe implants	LNA	*ex vivo *	Enhanced bone formation	[101]
miR-33	*Ldlr*^-/- ^mice	2'-F/MOE	s.c.	Increased serum HDL-C levels, regression of atherosclerosis	[108]
miR-33	DIO mice	LNA	i.v.	Increased serum HDL-C levels	[11]
miR-33	Chow-fed mice	n.d.	i.v.	Increased serum HDL-C levels	[129]
miR-380-5p	Orthotopic mouse model of neuroblastoma	LNA, 2'-F/MOE	i.p.	Decreased tumor growth	[130]
miR-182	Mouse model of arthritis	LNA	*ex vivo *	Amelioration of arthritis	[54]
miR-29c	db/db mice	2'-*O*-Me	i.p.	Reduced albuminuria and kidney mesangial matrix accumulation	[131]
miR-208a	Hypertensive rats	LNA	i.v.	Improved cardiac function and survival during heart failure	[102]

In an orthotopic xenograft model of metastatic breast cancer, 4T1 cells were implanted into the mammary fat pad of mice and miR-10b was targeted by antagomir-10b to investigate the effects of the primary tumors and their metastatic capacity [123]. This is a very aggressive metastasis model and, therefore, antagomir treatment was initiated already at day 2 after implantation to interfere with early stages of metastasis. The antagomir compound was administered i.v. twice weekly for three weeks (50 mg/kg) and mice were analyzed at day 28. Treatment did not reduce primary mammary tumor growth of 4T1 cells, whereas a striking suppression in the formation of lung metastases was observed (86% reduction of pulmonary metastases). The inhibition of miR-10b was validated by qRT-PCR and de-repression of the direct miR-10b target Hoxd10. Specificity was demonstrated by unaltered levels of miR-9 and miR-21 that are reported to be up-regulated in breast tumors and unchanged miR-10a, which differs by only 1 nt compared to the mature miR-10b sequence. In addition, a miR-10b sponge approach phenocopied the antagomir data, whereas no effect of miR-10b inhibition on lung metastases from disseminated cells (tail vein injected 4T1 cells) was observed, suggesting that miR-10b is not involved in late stage metastasis. The tolerability and toxicity of the antagomir treatment was assessed by several parameters, including behavior, body, lung and heart weight, respectively, white blood cell and lymphocyte count, histopathological investigations of steatosis, inflammation, necrosis, fibrosis and biliary changes. The most noticeable changes were the lowering of white blood cells and lymphocytes for miR-10b antagomir and as a suggested antagomir class effect; increased liver and spleen size, and elevated serum levels of bilirubin, ALT and AST [123].

Recently, miR-103 and miR-107 were shown to directly regulate insulin sensitivity *in vivo *[128]. This miRNA family was up-regulated in the liver of diet-induced obese and ob/ob mice, which led to decreased insulin sensitivity and enhanced hepatic glucose production. Cholesterol-conjugated antagomir-103 was applied to investigate the function of miR-103/107 in diabetes and administered via the tail vein on two consecutive days (15 mg/kg/dose). Targeting of miR-103/107 was demonstrated by Northern blot analysis, qRT-PCR and de-repression of the direct target Caveolin-1, whereas specificity was shown using mismatched and scrambled antagomirs. Silencing of miR-103/107 lowered plasma glucose levels in obese but not in wild-type mice, and improved glucose homeostasis and insulin sensitivity. Furthermore, over-expression or antagomir mediated silencing of miR-103/107 in diet-induced obese mice lacking Caveolin-1 demonstrated a central role of Caveolin-1 in mediating the miR-103/107 effects on glucose tolerance and insulin sensitivity [128].

Systemic lupus erythematosus (SLE) is a chronic autoimmune disease, in which a combination of genetic predisposition and possible environmental factors triggers an immune response directed against ubiquitous, mostly intranuclear, self-antigens. Antibody production by B cells and abnormal antibody-independent B and T cell functions imply that B and T cells are important in the pathogenesis of SLE (reviewed in [132,133]). The B6.Sle123 mouse strain bears three lupus susceptibility loci and develops an autoimmune syndrome that strongly resembles human lupus disease, characterized by autoantibody production, lymphosplenomegaly and glomerulonephritis. In a recent study, miR-21 was found to be up-regulated in B and T cells of B6.Sle123 mice [115], consistent with findings in another genetic mouse model of lupus as well as in human lupus CD4^+ ^T cells and B cells [134,135]. Silencing of miR-21 by i.p. delivered, unconjugated 8-mer seed-targeting antimiR-21 reversed splenomegaly, one of the cardinal manifestations of autoimmunity in B6.Sle123 mice and de-repressed PDCD4 expression *in vivo*. In addition, antimiR-21 treatment altered CD4^+/^CD8^+ ^T cell ratios towards those of the non-autoimmune control mice and reduced Fas receptor-expressing B cells, suggesting that miR-21 plays a critical role in regulating autoimmune responses in lupus. Furthermore, these findings imply that tiny seed-targeting LNAs can be used to inhibit miRNAs in peripheral lymphocytes *in vivo *and that pharmacological inhibition of miR-21 by an 8-mer antimiR-21 can alter the course of a systemic autoimmune disease in lupus mice [115].

The miR-208a/b family and miR-499, designated as MyomiRs, are located in the introns of three myosin genes, Myh6, Myh7, and Myh7b, respectively, and play critical roles in the control of pathological cardiac hypertrophy, heart failure and myocardial infarction in humans and mouse models of heart disease [10,136]. Genetic deletion of miR-208 in mice showed no phenotype at baseline, whereas in response to cardiac stress, miR-208 knockout mice showed virtually no cardiomyocyte hypertrophy or fibrosis [137,138]. In a recent study, Montgomery *et al. *[102] investigated the cardioprotective effect of miR-208a loss-of-function in hypertensive rats. Therapeutic silencing of miR-208a by subcutaneously (s.c.) delivered LNA-modified antimiR-208a led to potent and sustained silencing of miR-208a in the rat heart. Notably, the antimiR treatment prevented pathological myosin switching and cardiac remodeling during hypertension-induced heart failure in Dahl hypertensive rats, and resulted in improved cardiac function, overall health and survival. These data highlight the potential of antimiR-based approaches to pharmacologically inhibit cardiac miRNAs and strongly imply miR-208 as a therapeutic target for treatment of heart disease [102].

Perturbations in cholesterol homeostasis and lipid metabolism are associated with several life-threatening diseases, such as atherosclerosis, type II diabetes and metabolic syndrome. In 2010, a number of independent studies reported that miR-33a, which is embedded within an intron of the sterol regulatory element-binding protein-2 (SREBP2) gene, targets the ATP-binding cassette transporter A1 (ABCA1), an important regulator of high-density lipoprotein (HDL) synthesis and reverse cholesterol transport, for post-transcriptional repression [11,12,129,139,140]. Interestingly, another member of the miR-33 family, miR-33b, is found within an intron of the SREBP-1c gene in human and primates, whereas mice only have one miR-33 isoform corresponding to miR-33a [11]. The mature sequences of miR-33a and miR-33b differ by only two nucleotides and share the same seed region, implying that the two miR-33 family members have overlapping targets, and, thus, redundant biological functions, including regulation of cholesterol efflux in cells. Three *in vivo *studies have used antimiR oligonucleotides to probe the functions of miR-33 in cholesterol homeostasis in the mouse. Marquart *et al. *[129] delivered antimiRs intravenously (5 mg/kg/dose on three consecutive days) and showed increased ABCA1 expression and HDL-cholesterol levels in serum 12 days after administration, whereas Najafi-Shoushtari *et al. *[11] injected a LNA-modified antimiR-33 i.v. at a dose of 20 mg/kg for three consecutive days, which resulted in efficient inhibition of miR-33 and concomitant increase of HDL-C by 25% in the mouse serum. More recently, a third *in vivo *study targeting miR-33 was reported, in which low-density lipoprotein (LDL) receptor knockout mice with established atherosclerotic plaques were treated with s.c. delivered 2'F/MOE antimiR for four weeks (two s.c. injections of 10 mg/kg the first week followed by weekly injections of 10 mg/kg) [108]. Treatment of Ldlr^-/- ^mice with antimiR-33 led to elevated circulating HDL-C levels and enhanced reverse cholesterol transport to the plasma, liver and feces. Moreover, several markers of atherosclerotic plaque stability were increased, which was consistent with plaque regression and lesion remodelling in antimiR-33 treated mice. Importantly, this study showed that antimiR-33 oligonucleotides are able to penetrate the atherosclerotic lesion to reach plaque macrophages, in which they can enhance ABCA1 expression and cholesterol removal [108]. Together, these studies demonstrate that pharmacological inhibition of miR-33 *in vivo *by antimiR-33 oligonucleotides raises circulating HDL-C levels, enhances reverse cholesterol transport and regresses atherosclerosis, implying that therapeutic silencing of miR-33 could be a useful strategy for the treatment of cardiovascular disease.

## Therapeutic targeting of microRNA-122 for treatment of hepatitis C virus infection

Hepatitis C virus (HCV) infection is a leading cause of liver disease worldwide with over 180 million infected individuals who are at greatly increased risk of developing liver failure and hepatocellular carcinoma (HCC). The current standard therapy, which combines pegylated interferon-α with ribavirin provides sustained virologic response rates in only about 50% of patients and is also associated with many side effects [141]. New targeted HCV therapies, including viral polymerase and protease inhibitors have yielded encouraging results, but the emergence of viral escape mutations during such therapies requires a combination with other HCV drugs to tackle viral resistance [142]. By comparison, therapeutic approaches that target essential host functions for HCV may provide a high barrier to resistance and, thus, could provide an alternative strategy for the development of new HCV therapeutics. The liver-expressed miR-122 binds to two closely spaced miR-122 target sites in the 5' non-coding region (NCR) of the HCV genome, resulting in up-regulation of viral RNA levels [143]. This unusual interaction was first described by Peter Sarnow in 2005 [143], and was subsequently confirmed by several reports [144-146], implying that miR-122 is an essential host factor for HCV RNA accumulation in infected liver cells. Notably, inhibition of miR-122 by antimiR oligonucleotides leads to rapid loss of HCV RNA in cultured liver cells, which makes miR-122 an attractive therapeutic target for antiviral intervention [143,146]. In a recent study, Machlin *et al. *[147] investigated the contributions of the two miR-122 molecules by assessing the effects of miR-122 point mutations on HCV viral RNA abundance. The data from stepwise mutational analyses suggest a model for an oligomeric miR-122-HCV complex in which one miR-122 molecule binds to the 5' terminus of the HCV RNA with 3' overhanging nucleotides masking the 5' terminal sequences of the HCV genome. These findings suggest that miR-122 protects the 5' terminal viral sequences from nucleolytic degradation or from inducing innate immune responses to the RNA terminus [147].

Besides its role in modulating cholesterol homeostasis and promoting HCV RNA abundance, miR-122 has also been suggested to be important for maintaining liver cell identity and reported to be down-regulated in HCC [148-150]. Loss of miR-122 expression in HCC was shown to be associated with poor prognosis, acquisition of an invasive phenotype and with intrahepatic metastasis [150-152]. The tumor-suppressive effects of miR-122 have been linked to several direct miR-122 targets implicated in HCC tumorigenesis, such as cyclin G1, RHOA and the metalloprotease ADAM17. Interestingly, other studies have reported that miR-122 expression is either maintained or increased in HCV-associated HCC [150,153]. Moreover, Varnholt *et al. *[153] observed strong up-regulation of miR-122 in an extended sample set of HCV-induced dysplastic nodules and HCCs, which implies that the role of miR-122 in HCV-derived HCCs is different compared to that in HCCs of non-HCV etiologies. While further studies are needed to establish the potential risks associated with long-term therapeutic silencing of miR-122, it is important to note that short-term inhibition of miR-122 in rodents and non-human primates was shown to be reversible [90,96], and furthermore, that the duration of treatment of HCV-infected patients with an antimiR-122 is expected to be limited.

Several studies have reported on pharmacological inhibition of miR-122 in mice using antimiR oligonucleotides [77,90,96,100,104,106]. We have previously shown that potent miR-122 antagonism can be achieved in rodents and non-human primates using a high-affinity 15-mer LNA-modified antimiR-122. In this study, systemic delivery of unconjugated, saline-formulated antimiR-122 resulted in efficient sequestration of miR-122 leading to a dose-dependent and long-lasting decrease of serum cholesterol levels in mice and African green monkeys without any evidence for acute or subchronic toxicities in the study animals [90]. Moreover, this antimiR oligonucleotide was highly potent in inhibiting HCV RNA accumulation in Huh-7 cells harboring the HCV-N replicon NNeo/C-5B [90]. More recently, we assessed the potential of miR-122 antagonism as a new anti-HCV therapy in chimpanzees with chronic HCV infection [107]. In this study, four chimpanzees infected with HCV genotype 1 were treated with i.v. injections of the 15 nt LNA-antimiR-122 on a weekly basis for 12 weeks, followed by a treatment-free period of about 12 weeks after dosing. Treatment of the HCV-infected chimpanzees led to long-lasting suppression of HCV viremia with no evidence for viral resistance or side effects in the treated animals. Furthermore, transcriptional profiling and histopathology of liver biopsies demonstrated de-repression of target mRNAs with canonical miR-122 seed sites, down-regulation of interferon-regulated genes and improvement of HCV-induced liver pathology [107]. The long-lasting suppression of HCV viremia without viral rebound implies that the antimiR-122 approach has a high barrier to viral resistance. Furthermore, the fact that both miR-122 seed sites are conserved in all HCV genotypes suggests that the antiviral effect of antimiR-122 will be genotype-independent, which was recently confirmed [154].

Indeed, this antimiR-122 compound, termed miravirsen, is the first miRNA-targeted drug to enter human clinical trials. Data from phase 1 single (up to 12 mg/kg) and multiple ascending dose (up to five doses of 5 mg/kg) safety studies in 77 healthy volunteers showed that miravirsen is well tolerated, has an attractive pharmacokinetic profile and clear dose-dependent pharmacology. Importantly, no dose limiting toxicities were identified [155,156]. In September 2010, Santaris Pharma A/S advanced miravirsen into a phase 2a trial to assess the safety, tolerability, pharmacokinetics and antiviral activity of miravirsen in treatment-naïve patients with chronic HCV genotype 1 infection [155,156]. In this multiple ascending dose study patients were enrolled sequentially to one of three cohorts (nine active and three placebo per cohort) and miravirsen was administered at doses of 3, 5 or 7 mg/kg as a total of five weekly subcutaneous injections over 29 days. Treatment with miravirsen provided robust, dose-dependent anti-viral activity with a mean reduction of two to three logs from baseline in HCV RNA (log_10 _IU/mL) that was maintained for more than four weeks after the last dose of miravirsen. Notably, four out of nine patients treated at the highest dose (7 mg/kg) became HCV RNA undetectable during the study [155,156]. No serious adverse events were observed and only mild and infrequent adverse events, such as headache, coryza and diarrhea were reported. Furthermore, there were no clinically significant changes in safety tests, vital signs or electrocardiograms [155,156]. As expected, pharmacological inhibition of miR-122 in HCV patients resulted in decreased levels of serum cholesterol, apoA and apoB. Taken together, these data indicate that miravirsen given as a four-week monotherapy to HCV patients provides long-lasting suppression of viremia, has a high barrier to viral resistance and is well tolerated in patients with chronic HCV infection.

## Conclusions

The challenge of unravelling the myriad roles of hundreds of miRNAs in many developmental and cellular processes as well as in human disease pathogenesis calls for continuous development of robust computational and experimental approaches for studying miRNA functions in cell culture and *in vivo*. Inhibition of miRNA function by chemically modified antimiR oligonucleotides has become an important and widely used approach in miRNA loss-of-function studies and enables inhibition of both single miRNAs and entire miRNA seed families. Despite recent advances in the design and use of antimiRs, experiments that seek to inhibit miRNA function are associated with several possible pitfalls when antimiR mediated miRNA inhibition is assessed. Furthermore, the use of antimiR oligonucleotides as tools in miRNA loss-of-function studies or as therapeutic modalities carries the inherent risk of affecting RNA species other than the intended miRNA target. Hence, adequate assessment of the functional effects after miRNA inhibition and the physiological repercussions of long-term miRNA antagonism *in vivo*, as well as understanding the potential off-target effects resulting from unwanted interactions between the antimiR oligonucleotide and endogenous nucleic acids, are of key importance for antimiR-based miRNA loss-of-function studies and for the development of miRNA therapeutics.

Efficient *in vivo *delivery of antimiR oligonucleotides is another critical factor for their successful use *in vivo *and for the development of miRNA-based therapeutic modalities. Many peripheral tissues can be effectively targeted by systemically delivered chemically modified antimiR oligonucleotides, which show good pharmacokinetic properties and tissue uptake along with high stability in blood and tissues *in vivo*. A number of alternative strategies for delivery of antisense oligonucleotides and siRNAs are being pursued and these could also be applied to antimiRs. For example, ligands for specific cell surface receptors capable of being internalized can be conjugated to oligonucleotides, thereby facilitating both cellular uptake and cell type-specific delivery. Nevertheless, recent findings that unconjugated, saline-formulated antimiR oligonucleotides can be used in miRNA silencing *in vivo *suggest that antimiRs are useful tools for validating disease-associated miRNA targets in animal disease models. Furthermore, the high potency and metabolic stability of chemically modified antimiRs, and the lack of acute and subchronic toxicities in rodents and non-human primates highlights the potential of antimiRs in the development of novel therapeutic modalities based on disease-associated miRNAs. Indeed, recent data from the first phase 2 study in patients with chronic HCV genotype 1 infection treated with the LNA-modified antimiR-122 drug miravirsen showed that this compound was well tolerated and provided long-lasting suppression of viremia in HCV-infected patients.

## Abbreviations

2'-F: 2'-fluoro; 2'-MOE: 2'-*O*-methoxyethyl; 2'-*O*-Me: 2'-*O*-methyl; ABCA1: ATP-binding cassette transporter A1; ALT: alanine aminotransferase; antimiRs, antisense oligonucleotides that inhibit miRNA function; AST: aspartate aminotransferase; CNS: central nervous system; DIO: diet induced obesity; EAE: experimental autoimmune encephalomyelitis; HCC: hepatocellular carcinoma; HCV: hepatitis C virus; HDL: high-density lipoprotein; i.p., intraperitoneal; i.v., intravenous; LDL: low-density lipoprotein; LNA: locked nucleic acid; miRISC: miRNA-induced silencing complex; miRNA: microRNA; NCR: non-coding region; PS: phosphorothioate; RNA-Seq: RNA sequencing; s.c., subcutaneous; SLE: systemic lupus erythematosus; SREBP2: sterol regulatory element-binding protein-2; T_m_: melting temperature; UTR: untranslated region.

## Competing interests

AP, ML, SO and SK are employees of Santaris Pharma, a clinical stage biopharmaceutical company that develops RNA-directed therapeutics. JS has no competing interests.

## Authors' contributions

JS, AP and SK drafted the manuscript with input from SO and ML. All authors reviewed and approved the final manuscript.
